# Feasibility to estimate mean systemic filling pressure with inspiratory holds at the bedside

**DOI:** 10.3389/fphys.2022.1041730

**Published:** 2022-11-29

**Authors:** Marije Wijnberge, Jos R. C. Jansen, Michael R. Pinsky, Robert B. Klanderman, Lotte E. Terwindt, Joachim J. Bosboom, Nikki Lemmers, Alexander P. Vlaar, Denise P. Veelo, Bart F. Geerts

**Affiliations:** ^1^ Amsterdam UMC Location Academic Medical Center, Department of Anesthesiology, Amsterdam, Netherlands; ^2^ Amsterdam UMC Location Academic Medical Center, Department of Intensive Care Medicine, Amsterdam, Netherlands; ^3^ Leiden University Medical Center, Department of Intensive Care Medicine, Leiden, Netherlands; ^4^ Department of Critical Care Medicine, University of Pittsburgh, Pittsburgh, PA, United States; ^5^ Healthplus.ai, Amsterdam, Netherlands

**Keywords:** hemodynamics, venous return, mean circulatory filling pressure, physiology, volume status, Guytonian model, vascular compliance

## Abstract

**Background:** A decade ago, it became possible to derive mean systemic filling pressure (MSFP) at the bedside using the inspiratory hold maneuver. MSFP has the potential to help guide hemodynamic care, but the estimation is not yet implemented in common clinical practice. In this study, we assessed the ability of MSFP, vascular compliance (Csys), and stressed volume (Vs) to track fluid boluses. Second, we assessed the feasibility of implementation of MSFP in the intensive care unit (ICU). Exploratory, a potential difference in MSFP response between colloids and crystalloids was assessed.

**Methods:** This was a prospective cohort study in adult patients admitted to the ICU after cardiac surgery. The MSFP was determined using 3–4 inspiratory holds with incremental pressures (maximum 35 cm H_2_O) to construct a venous return curve. Two fluid boluses were administered: 100 and 500 ml, enabling to calculate Vs and Csys. Patients were randomized to crystalloid or colloid fluid administration. Trained ICU consultants acted as study supervisors, and protocol deviations were recorded.

**Results:** A total of 20 patients completed the trial. MSFP was able to track the 500 ml bolus (*p* < 0.001). In 16 patients (80%), Vs and Csys could be determined. Vs had a median of 2029 ml (IQR 1605–3164), and Csys had a median of 73 ml mmHg^−1^ (IQR 56–133). A difference in response between crystalloids and colloids was present for the 100 ml fluid bolus (*p* = 0.019) and in a post hoc analysis, also for the 500 ml bolus (*p* = 0.010).

**Conclusion:** MSFP can be measured at the bedside and provides insights into the hemodynamic status of a patient that are currently missing. The clinical feasibility of Vs and Csys was judged ambiguously based on the lack of required hemodynamic stability. Future studies should address the clinical obstacles found in this study, and less-invasive alternatives to determine MSFP should be further explored.

**Clinical Trial Registration:** ClinicalTrials.gov Identifier NCT03139929.

## Introduction

At present, a decade after it became possible to estimate mean systemic filling pressure (MSFP) at the bedside, the parameter has not yet been implemented in (routine) clinical care. MSFP is considered the combined upstream pressure that drives blood flow into the right atrium, and MSFP allows the calculation of additional hemodynamic parameters such as the driving pressure for venous return (VRdp), stressed volume (Vs), and total systemic vascular compliance (Csys) (Maas et al., 2012a). Vs provides information on the effective circulating volume, a hemodynamic variable that is missing in current clinical practice. MSFP has helped to better understand the effects of vasopressors, propofol, and hyperoxia (Maas et al., 2013; de Wit et al., 2016; Helmerhorst et al., 2017; Adda et al., 2021). MSFP and the derived parameters could potentially be beneficial to guide hemodynamic care in patients admitted to the intensive care unit (ICU) (Rothe, 1993; Vos et al., 2020; Persichini et al., 2022).

MSFP can be determined in sedated and ventilated patients by extrapolating central venous pressure (CVP) versus cardiac output (CO) at different ventilatory plateau pressures during inspiratory holds (Wijnberge et al., 2018). Previous studies showed MSFP to predict fluid loading responsiveness (Guerin et al., 2015; Cooke et al., 2018). Although MSFP sounds promising, studies describing clinical guidance based on MSFP and the derived parameters are lacking (Vincent and Pinsky, 2018). Also, in previous MSFP studies (Keller et al., 2011; Maas et al., 2012b; Maas et al., 2012c), colloids were used, limiting the clinical translatability of results and feasibility, since in ICU patients, crystalloids are the preferred choice of fluids (Maas et al., 2012a).

In this study, our first aim was to assess the ability of MSFP, Csys, and Vs to track two fluid boluses. Our second aim was to assess the feasibility of the clinical implementation of MSFP in the ICU. In exploratory, as a third aim, a potential difference in response between colloids and crystalloids was assessed. As the intravascular half-life for crystalloids is around 20–40 min and for colloids 2–3 h, we hypothesized a difference in the delta MSFP after a fluid bolus (Hahn and Lyons, 2016). If present, this would question the use of crystalloids for Csys and Vs determinations.

## Materials and methods

### Participants

This was a prospective cohort study in post-surgical patients after coronary artery bypass grafting (CABG). The study is written according to the Strobe guidelines for cohort studies and was conducted in accordance with the Declaration of Helsinki (von Elm et al., 2007; Cuschieri, 2019). The study took place at the ICU of the Amsterdam University Medical Centers, located at the Academic Medical Center (AMC). The study was approved by the Medical Ethics Committee (NL5531.018.15) and was registered at clinicaltrials.nl before the start of the study (NCT03139929). Written informed consent was obtained prior to surgery. Patients were included between 2017 and 2019. Adult patients (>18 years old) scheduled to undergo elective CABG surgery were included. The exclusion criteria before surgery were morbid obesity (BMI > 40), right- or left-sided heart failure, significant valvular regurgitation or stenosis, arrhythmias, intra-cardiac shunts, symptomatic peripheral vascular disease, and symptomatic pulmonary disease.

During surgery, anesthesia was provided as per routine care. At the end of surgery, noradrenalin, propofol, and/or sufentanil were continued for transport to the ICU. The exclusion criteria at the ICU were a contraindication for fluid loading and persistent hemodynamic instability. Hemodynamic instability was defined as a persistent mean arterial pressure (MAP) below 55 mmHg, a cardiac index below 1.5 L/min/m^2^ or patients in which the MAP remained highly fluctuating (delta 40 mmHg in 10 min) after optimizing initial treatment. A maximum of 1 hour was allowed for patients to fulfil the hemodynamic stability criteria after arrival in the ICU. During study measurements, no alterations in the respiratory rate, positive end expiratory pressure (PEEP), fraction of inspired oxygen (FiO_2_), and position of the patient were allowed. Also, the rates of anesthetic, analgesic, and vasoactive drugs were set before the start of the study and could not be altered during study measurements.

### Study measurements

Study measurements were performed by a dedicated study team consisting of one member to control the ventilator (inspiratory holds), one member for circulation (administering fluid bolus), one member as the annotator, and one as the supervising ICU consultant.

Arterial blood pressure (ABP) was monitored *via* a catheter in the radial artery, and CVP was monitored *via* a catheter inserted into the right internal jugular vein. Both were connected to a pressure transducer, and both pressure transducers were referenced to the intersection of the anterior axillary line and the fifth intercostal space. Beat-to-beat CO was obtained by Modelflow pulse contour analysis (de Wilde et al., 2007). Measurements were recorded at a sample frequency of 100 Hz and 0.2 mmHg of resolution.

MSFP was measured employing successive inspiratory holds as previously published (Maas et al., 2009; Maas et al., 2012a). In short, four inspiratory holds were executed at different pressure levels, namely, 5, 15, 25, and 35 cmH_2_O above PEEP. The CVP and CO data at those inspiratory holds were fitted by linear regression. A previous animal study demonstrated three holds sufficient to reliably assess MSFP (Maas et al., 2011). Therefore, an MSFP measurement was judged successful if at least three holds were performed. If the third inspiratory hold (25 cmH2O above PEEP) resulted in a significant decrease in MAP (defined as a MAP below 50 mmHg), the fourth inspiratory hold (35 cmH_2_O above PEEP) could be omitted as decided by the supervising ICU consultant.

**FIGURE 1:**
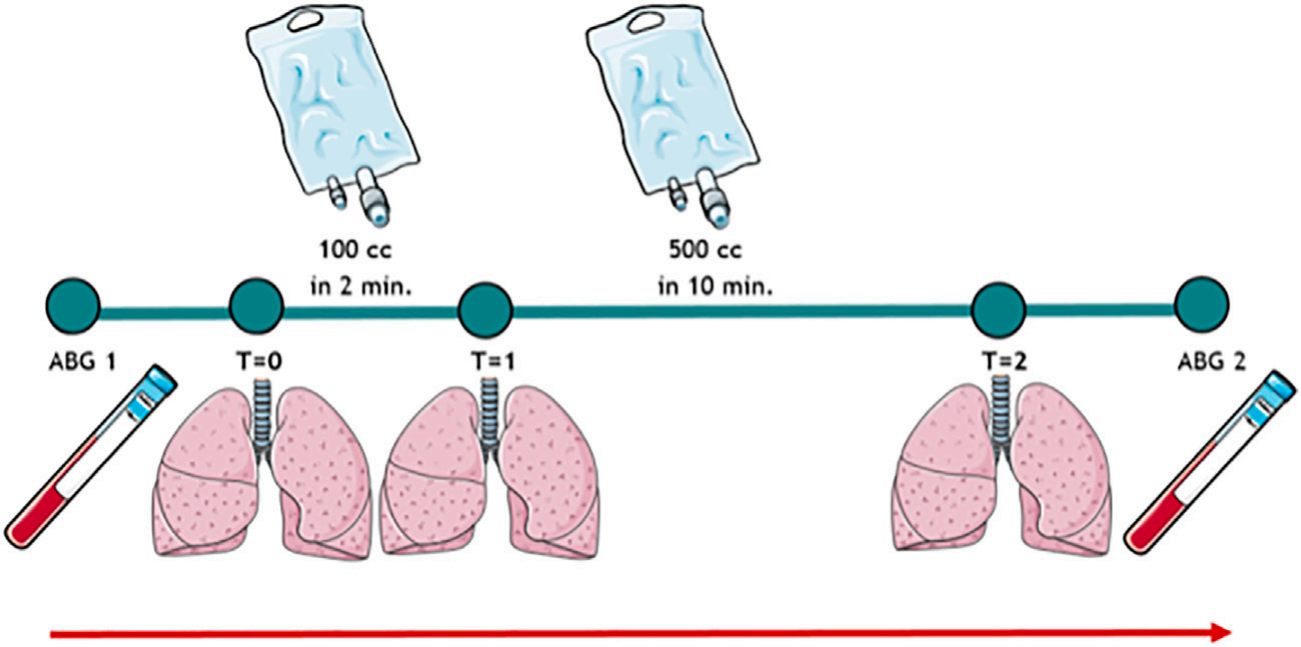
Visual study protocol. ABG, arterial blood gas.

For this study, MSFP was measured at three timepoints: at baseline (T = 0), after 100 ml of fluid loading (T = 1), and after a second bolus of 500 ml (T = 2), Figure 1. Both fluid bolus sizes were chosen based on previous studies (Maas et al., 2012a; Smorenberg et al., 2018) and were given at the same infusion speed of 50 ml/min. The supervising ICU consultant could terminate fluid infusion if the ABP increased excessively. No blood pressure cut-off values were defined as the allowed maximum systolic blood pressure (SBP) could differ per patient.

To assess the effects of the type of fluid on MSFP, 50% of the patients who fulfilled hemodynamic stability criteria prior to start of the study were randomized between crystalloid (Sterofundin, BBraun) or colloid infusion (Tetraspan, BBraunn) (Hahn and Lyons, 2016)

### Outcomes

MSFP was determined as explained previously, by extrapolating CO versus CVP at different inspiratory plateau pressures to CVP is zero. CVP was used as a surrogate for right atrial pressure. The driving pressure for venous return (VRdp) was defined as MSFP-CVP. Venous return (VR) was defined as VRdp divided by resistance to venous return (RVR). RVR is the reciprocal of the slope of the VR curve, or RVR = (MSFP-CVP)/CO. The total systemic vascular resistance (Rsys) was calculated as the ratio between the pressure difference of MAP and CVP with CO (Jansen et al., 2010; Maas et al., 2012a; Maas et al., 2012b).

With MSFP measured before and after fluid administration, a pressure-volume relationship could be constructed. Csys is the slope of this relation, or delta volume/delta MSFP (Maas et al., 2012a). Csys = fluid bolus/(MSFP after bolus–MSFP before bolus). Vs = Csys x MSFP.

Since Vs and Csys may vary widely if sympathetic tone or blood flow distribution varies, hemodynamic stability was required during the two volume challenges. Hemodynamic instability during the study was defined as a change in heart rate exceeding 10 beats per minute between two time points (T = 0, T = 1, and T = 2), a decrease in MAP despite fluid administration, or a change in the respiratory rate.

Protocol deviations, to assess feasibility, were defined as any deviation from the study protocol. The supervising ICU consultant was asked to clarify the rationale for the protocol deviation.

### Statistical analysis

Continuous data are presented as means with standard deviations when normally distributed or as medians with interquartile ranges (IQR 25–75th) when the data were not normally distributed. The distribution was assessed visually based on Q–Q plots and histograms. Categorical data are presented as frequencies with percentages. The paired t-tests or the non-parametric Wilcoxon signed-rank test were used for comparison of hemodynamic variables on T = 0, T = 1, and T = 2. The independent t-test or the non-parametric Mann–Whitney *U* test were used for differences between colloid and crystalloid groups. A post hoc analysis was performed to correct for patients who did not receive the total amount of 500 ml during the second fluid bolus. The post hoc analysis was performed by dividing the planned fluid administration (=500 ml) by the actual administered fluids (in ml) and multiplying with the delta MSFP.

A *p* value of <0.05 was considered to indicate significance. All analyses were performed using MATLAB version 14 and SPSS version 28.

### Sample size analysis

To detect the 500 ml bolus, a sample size of seven patients was calculated to have 90% power to detect a difference in Vs means of 500 ml, assuming a standard deviation of 400 ml, using a paired *t*-test with a 0.05 two-sided significance level.

To detect the 100 ml bolus, a sample size of 38 patients was calculated to have 90% power to detect a difference in Vs means of 100 ml, assuming a standard deviation of 210 ml, using a paired *t*-test with a 0.05 two-sided significance level.

Assuming a drop-out rate of 10%, a sample size of 42 patients was calculated based on the first 100cc-fluid bolus, between T = 0 and T = 1. As this was the smallest fluid bolus, it required the largest number of patients.

Sample sizes were calculated using nQuery Advanced, version 8.5.1.

## Results

### Study population

For this prospective cohort study, 121 patients were assessed for eligibility, of these 44 patients had undergone solely CABG surgery and were enrolled in the ICU. A total of 20 patients completed the trial. The exclusion of 24 patients before the start of study measurements at ICU was because of hemodynamic instability (*n* = 18) or because of logistic reasons (*n* = 6), e.g., night-time or incomplete study team, Supplementary Material S1. The median age was 65 years and 100% were men. Table 1 and Supplementary Material S2 show the baseline characteristics. A total of four out of 20 (20%) patients were judged fluid loading responsive (FLR), defined as a 12% increase in CO after the second fluid bolus (Cherpanath et al., 2013). No serious adverse events occurred in both the colloid and crystalloid groups.

**TABLE 1:** Baseline characteristics.

	*n* = 20
Age	66 ± 8.7
Men	20 (100%)
Height (in cm)	178.7 ± 6.5
Weight (in kg)	88.6 ± 11.2
BMI	27.8 ± 3.7
ASA I	0
ASA II	0
ASA III	16 (80%)
ASA IV	4 (20%)
Medical history	
Hypertension	9 (45%)
Heart failure	0
COPD	0
OSAS	1 (5%)
Obesity	4 (20%)
Diabetes	6 (30%)
Renal insufficiency	1 (5%)
Hypothyroidism	2 (10%)
Relevant medication	
Beta-blocker	16 (80%)
Type of surgery	
CABG on pump	20 (100%)
Surgery duration (min)	252.5 (237.8–324.5)
	
CPB duration (min)	87.5 (72.0–132.3)
Aortic clamp time (min)	61.5 (45.0–73.5)
TEE after cardiac bypass by cardiac anesthesiologists	
Good LVF and RVF	18 (90%)
Moderate LVF	2 (10%)

ASA, American Society of Anesthesiologists; BMI, body mass index; COPD, chronic obstructive pulmonary disease; OSAS, obstructive sleep apnea syndrome; CABG, coronary artery bypass grafting; CPB, cardiopulmonary bypass; TEE, transesophageal echocardiography; LVF, left ventricular function; RVF, right ventricular function; Min, minutes. Continuous data are presented as a mean with standard deviation (±) or as a median with interquartile ranges (IQR 25th–75th). Categorical data are presented as numbers with percentages (%). The ASA classifications were as follows: 1) a healthy person, 2) a patient with mild systemic disease, 3) a patient with severe systemic disease, and 4) a patient with severe systemic disease that is a constant threat to life.

### Mean systemic filling pressure

Providing a fluid bolus increased MSFP as expected, with mean MSFP at T = 0 of 20.08 mmHg ±3.77, at T = 1 of 21.88 mmHg ±4.72 and at T = 2 of 26.82 mmHg ±5.58, Table 2.

**TABLE 2:** Hemodynamic changes after two fluid boluses.

	T = 0	T = 1	T = 2	p1	p2
MSFP	20.08±3.77	21.88 ± 4.72	26.82 ± 5.58	**0.005**	**<0.001**
Fluid bolus		100 ± 0	465 ± 103		
		100 (100–100)	500 (425–500)		
HR	69 ± 11	68 ± 10	66 ± 9	**0.028**	**0.049**
MAP	72 ± 6	79 ± 7	88 ± 11	**<0.001**	**<0.001**
CO	5.25 ± 1.53	5.12 ± 1.26	5.40 ± 1.35	0.397	**0.003**
CVP	6.86 ± 2.62	7.24 ± 2.70	8.63 ± 3.35	**0.004**	**<0.001**
SVR	1158 (934–1450)	1322 (1067–1607)	1393 (1169–1617)	**0.025**	0.478
VRdp	13.22 ± 2.38	14.97 ± 3.50	18.19 ± 3.63	**0.012**	**<0.001**
RVR	2.53 (1.89–3.27)	2.78 (2.37–3.66)	3.38 (2.94–4.11)	**0.044**	**0.004**
Rsys	13.15 (9.45–16.62)	14.65 (11.33–17.90)	15.44 (11.35–19.46)	**0.004**	0.204
PPV	8.94 (7.72–11.48)	7.99 (5.69–10.51)	5.29 (3.26–8.33)	0.351	**<0.001**
SVV	7.96 (5.92–9.19)	5.98 (4.87–8.00)	3.35 (2.58–6.28)	**0.030**	**<0.001**

MSFP, mean systemic filling pressure. HR, heart rate. MAP, mean arterial pressure. CO, cardiac output. CVP, central venous pressure. SVR, systemic vascular resistance [80*(MAP-CVP)/CO]. VRdp: driving pressure for venous return (MSFP-CVP). RVR: resistance to venous return [(MSFP-CVP)/CO]. Rsys: total systemic vascular resistance (MAP-CVP)/CO. Data are presented as a mean with standard deviation (±) or as a median with IQR (25th–75th) depending on the normality. *p* values 1 and 2 are determined with paired t-tests or the non-parametric related-samples Wilcoxon signed-rank test, depending on normality of the data. p1 demonstrates timepoint 0 versus timepoint 1. p2 demonstrates timepoint 1 versus timepoint 2.

The bold values represent statistically significant differences

### Stressed volume and compliance

Sixteen patients (80%) fit the inclusion criteria for hemodynamic stability (i.e., stable heart rate and no change in the respiratory rate during the study period) and were used for Vs and Csys calculations, Table 3 and Supplementary Material S3.

**TABLE 3:** Protocol deviations + reasons.

Fluid administration	
Complete first bolus (100 ml)	20/20 (100%)
Complete second bolus (500 ml)	15/20 (75%)
Crystalloid complete second bolus	12/15 (80%)
Crystalloid infused	Mean 483.67 ml ± 92.78
	Median 500 IQR 500-500
Colloid complete second bolus	3/5 (60%)
Colloid infused	Mean 410.00 ml ± 124.50
	Median 500 IQR 275–500
Reason ceasing infusion	Considerable increase in blood pressure
ABP at which infusion was ceased	
Maximum SBP	169 mmhg ± 11.97
Δ SBP	47 mmhg ± 6.58
MAP	97 mmhg ± 4.10
Δ MAP	24 mmhg ± 4.24
Inspiratory holds	
5, 15, and 25 cmH_2_O above PEEP	20/20 (100%)
5, 15, 25, and 35 cmH_2_O above PEEP	10/20 (50%)
Reasons not performing the fourth hold	Considerable decrease in MAP during the third hold
Lowest MAP during the third hold	49 mmhg ± 5.81
Δ MAP during third hold	25 mmhg ± 5.78
Lowest CO during the third hold	2.27 l ± 0.95
Vs and Csys calculations	
Haemodynamic instability during the study	4/20 (20%)
Reasons	
Δ HR more than 10 bpm between two timepoints (T = 0, T = 1, and T = 2)	2/20 (10%)
Starting to trigger ventilator after the 35-mmHg hold	1/20 (5%)
Decrease in MAP after fluid administration	1/20 (5%)

Continuous data are presented as a mean with standard deviation (±) or a median with inter quartile ranges (IQR 25th–75th). Categorical data are presented as frequencies with percentages. SBP, systolic blood pressure. MAP, mean arterial pressure. PEEP, positive end expiratory pressure. CO, cardiac output. HR. heart rate. Vs, stressed volume. Csys, compliance. Δ = delta.

Since Vs after 100 ml of crystalloid did not consistently result in an increase in MSFP, it was judged not reliable to present mean/median Vs and Csys at T = 1, Supplementary Material S3. Following, T = 2 had a median of 2028.95 ml (IQR 1605.08–3163.51) and Csys at T = 2 had a median of 72.74 ml mmHg^−1^ (IQR 55.77–132.58). Corrected for body weight, this translates to a median Vs of 24.17 ml kg^−1^ (IQR 15.68–38.48) and a median Csys of 0.87 ml mmHg^−1^ kg^−1^ (IQR 0.54–1.49).

### Protocol deviations and clinical feasibility

Table 3 summarizes the protocol deviations and reasons. In all 20 patients (100%), the predefined minimum of three holds could be performed, thus in all patients, MSFP determination was possible. In 10 out of 20 patients (50%), a total of four holds could be executed (at 5, 15, 25, and 35 cmH_2_O above PEEP). The reason for not performing a fourth hold was a significant temporary decrease in MAP (mean lowest MAP 49 mmHg ±5.81, for less than 20 s) after the third hold.

In 5 out of 20 patients, the second fluid bolus (500 cc) was terminated before the total volume was infused because of a considerable increase in the systolic blood pressure (mean highest systolic blood pressure of 169 mmHg ±11.97).

Supplementary Material S4 summarizes this single-center experience concerning the feasibility of MSFP, Vs, and Csys calculations in the ICU.

### Exploratory analyses: colloid vs. crystalloid

In the dissecting type of fluids, the choice to cease the infusion of fluids between T = 1 and T = 2 was 3 out of 15 (20%) in the crystalloid group and 2 out of 5 (40%) in the colloid group.

**FIGURE 2:**
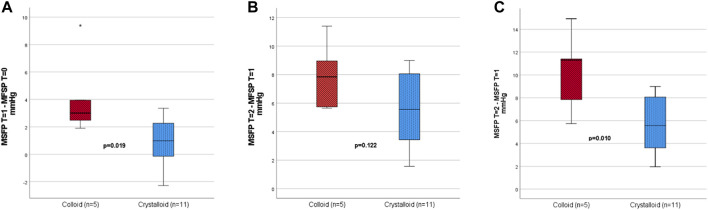
**(A)** Delta first fluid bolus, 100 ml; **(B)** delta second fluid bolus planned to be 500 ml, but in 40% of the colloid group the fluid infusion was ceased prematurely; and **(C)** post hoc analysis for a hypothetical delta MSFP if the total of 500 ml would have been administered. MSFP, mean systemic filling pressure. mmHg: millimetres of mercury. The thin vertical black stripe represents the minimum and maximum MSFP. The boxplot represents 25th–75th quartile. The horizontal thick black stripe represents median. *p* values for the independent *t*-test (first). Red and slash to the right = colloid. Blue and dots = crystalloid.

The independent t-test demonstrated a significant difference in the response on the first fluid bolus (100 ml) between crystalloids and colloids (*p* = 0.019), Figure 2. The paired t-test demonstrated the first colloid bolus resulted in a significant increase in MSFP (*p* = 0.038), whereas the first crystalloid bolus infusion did not (*p* = 0.110). For the second fluid bolus, no significant difference in delta MSFP between the types of fluids was found (*p* = 0.122), Figure 2. However, as the administered amount of fluid during the second bolus of colloid was lower than the administered bolus of crystalloid (Table 3), this was not a fair comparison. A post hoc analysis demonstrated that when the ceased fluid infusions were extrapolated to the planned 500 ml bolus, there was a significant difference in delta MSFP between crystalloid and colloid infusions, *p* = 0.01, Figure 2 and Supplementary Material S5.

## Discussion

This prospective study demonstrated the expected increase in MSFP, derived from 3–4 successive inspiratory holds after fluid loading. The clinical feasibility for MSFP determination was deemed sufficient, although labor intensive, but the clinical feasibility for Vs and Csys was judged ambiguously. The results of this study demonstrate the potential of MSFP and might partly explain why MSFP, Vs, and Csys, derived from inspiratory holds, are not yet widely implemented in clinical care.

### Previous studies

Our results are in line with previous studies demonstrating the effect of a fluid bolus on MSFP (Keller et al., 2011; Guerin et al., 2015). Although MSFP is thought central for the characterization of the circulation, the subsequent derived values such as Vs and Csys are subject to physiologic variability, though if accurate, of potentially greater clinical value (Rothe, 1993; Vos et al., 2020; Persichini et al., 2022).

In 1990, Vs was calculated to represent 30% ± 17% of total predicted blood in patients on the cardiac bypass for major vascular sugery (Magder and De Varennes, 1998). During hypothermic cardiac arrest, the cardiac bypass pump was turned off, and the blood that drained passively into a reservoir represented a mean Vs of 1,290 ml ±296, which equaled 20.2 ml kg^−1^ ±1.0 (Magder and De Varennes, 1998). This is close to the 19.5 ml kg^−1^ ±12.1 previously found in intact patients with the inspiratory hold technique (Maas et al., 2009), where Vs was a mean of 1,677 ml at baseline. In the present study, the median Vs was 2,028.95 ml at T = 2 (median 24.17 ml kg^−1^) after the total 600 ml fluid loading.

Previous clinical studies have found Csys to be 80 and 64.3 ml mmHg^−1^ (Maas et al., 2009; Maas et al., 2012a). In the present study, the median Csys was 72.74 ml mmHg^−1^. Thus, the present Vs and Csys values are in line with previous studies.

### Clinical feasibility

An MSFP measurement takes around 4–5 min. Estimating Vs and Csys thus requires at least 10 min (including fluid loading time) and it assumes the administered fluid is added to the stressed volume compartment (Hahn and Lyons, 2016) (Hahn et al., 2016). Interestingly, 100 ml of colloids did significantly increase MSFP, whereas 100 ml of crystalloids did not. A previous study also found the response on crystalloid variable in post-CABG patients (Sondergaard et al., 2019). Perhaps a capillary leak syndrome with endothelial glycocalyx shedding can partly explain our results, or that the expected transudation of crystalloid into the interstitium occurred rapidly in patients after cardiac surgery (Dekker et al., 2019). Furthermore, Vs and Csys calculations are based upon the assumption that the fluid administered adds directly to the stressed compartment (Vs) without alterations in the unstressed compartment. This assumption might not always be true if fluid administration also results in a shift of blood flow distribution across vascular beds with differing proportions of unstressed and stressed vascular volumes. We conclude a 100-ml bolus of crystalloids to be insufficient to reliably calculate Csys and Vs in this specific ICU population (Aya et al., 2016) (Aya et al., 2017)

Further scrutiny of the MSFP measurement, in the present study, shows that supervising ICU physicians were less inclined to allow the fourth hold (35 cmH_2_O) compared to previous studies because of (transient) hypotension (Maas et al., 2009; Maas et al., 2012a). Excluding the final 35 cm H_2_O inspiratory hold step in patients in whom a total of four holds could be executed, no significant change in the MSFP estimate was found; *p* = 0.696. This demonstrates that using high inspiratory hold levels may not be necessary. We noted that for executing the inspiratory holds, a deep level of sedation is necessary, exceeding the common level of sedation in ICU patients. Finally, although inspiratory holds have been shown to prevent postoperative pulmonary complications in non-ARDS patients (Hartland et al., 2015), they should not be performed in ARDS patients (Cavalcanti et al., 2017).

### Limitations

In only 20 patients, instead of the planned 42, study measurements could be performed. For the 20 patients, we were sufficiently powered (>90%) to detect a difference in Vs means of 500 ml, but a post hoc sample size analysis demonstrated this to reduce the power for detection of the 100 ml bolus to 56%. The majority of patients were excluded because they did not meet the hemodynamic stability criteria to start the study in the ICU. Our criteria could be too strict, or our cardiac surgery population more severely ill. Comparison with previous studies was not possible, as these numbers were not reported.

The colloid versus crystalloid analysis should be regarded as exploratory, but it could be used as a stepping stone for new trials. The post hoc analysis requires MSFP to increase linearly with infused fluids, as suggested by previous data (Maas et al., 2012a). Still, we cannot prove this linear association to be true for the presented data, so future studies should confirm our results.

The high number of protocol deviations in the study protocol might not solely describe clinical feasibility but can also illustrate that the supervising ICU consultants in the study hospital were more conservative.

In this study, we used Modelflow pulse contour to calculate CO. For MSFP, Vs and Csys absolute CO values are not necessary; trends are sufficient, Supplementary Material S6. However, for RVR, absolute values become relevant. Modelflow can be calibrated with thermodilution and echocardiography (Swan et al., 1970; de Vaal et al., 2005; Harvey et al., 2005; Tuggle, 2009).

An unintended but important finding is that all the patients studied were men. All the initially included women were deemed hemodynamically unstable in the ICU. Aiming to include women in order to obtain a study population that is reflective of the clinical population and in order to translate findings across genders remains important (Bybee and Stevens, 2013; Garcia et al., 2016; Gulati et al., 2021).

### What needs to happen to bring MSFP to clinical care, and what for?

MSFP determined with inspiratory holds is of great interest for research purposes, but based on this study, not yet ready for clinical use. Yet, less invasive alternatives for determining MSFP do exist (Wijnberge et al., 2018). The MSFP analogue is based on a model of the circulation, it is a calculation with CO, CVP, and MAP as input data. The MSFP analogue is much simpler to measure but was thought to suffer from greater inaccuracies, probably because of the assumptions in the calculation (Maas et al., 2012c; Meijs et al., 2022). The calculation uses standard arterial and venous compliances and resistances that might be inaccurate during acute disease states. However, a recent animal study concluded MSFP analogue to be the most reliable method to indirectly measure MSFP (Werner-Moller et al., 2019). The jury is still out, and this contradiction in results invites future research. A third method to estimate MSFP is based on the stop-flow principle, determined with a rapidly inflating cuff (halting blood flow) around the upper arm. A previous study demonstrated all three methods to track a fluid bolus (Maas et al., 2012c).

If MSFP determined with inspiratory holds is to become more commonly used, studies need to define the minimum number of inspiratory holds for accurate MSFP determination and should assess whether holds with lower plateau pressures also result in accurate MSFP values (Persichini et al., 2012; Guerin et al., 2015). Furthermore, if knowing accurate Vs and Csys is required, then defining the optimal fluid type (colloid vs. crystalloid) and the minimal volume challenge needed to determine Vs and Csys needs to be assessed. Being able to quickly and reliably calculate MSFP, Csys and Vs could be beneficial in guiding hemodynamic care in various types of patients (Rothe, 1993; Vos et al., 2020; Persichini et al., 2022). For example, in current sepsis resuscitation, first fluids are administered and subsequently (after >2 L of fluids is added in a normal sized adult patient), a vasopressor is started. Based on a Guytonian approach to the circulation, however, it would make more sense to start a vasopressor earlier in the treatment to recruit unstressed to the stressed volume (Adda et al., 2021; Persichini et al., 2022). Recruiting unstressed to stressed volume is an important survival mechanism of the human body. Measuring MSFP, Vs, and Csys might lead to a reduction in the total amount of fluids administered for resuscitation (Ospina-Tascón et al., 2020; Persichini et al., 2022). Despite the use of invasive hemodynamic monitoring options available in the ICU, we still lack direct and repetitive estimation of the effective circulating volume (Vs). Working with MSFP might enable us to go beyond fluid loading responsiveness and help us better understand the physiology during various clinical scenarios (Crozier et al., 2015; Moller et al., 2019; Persichini et al., 2022). Future studies should study whether adding MSFP, Vs, and Csys to our clinical arsenal actually result in improved patient outcomes.

## Conclusion

Mean systemic filling pressure, estimated with inspiratory holds, behaves predictably conform known physiological principles. Clinical feasibility for Csys and Vs calculations was judged ambiguously based on the lack of required hemodynamic stability and on the assumption of administered fluids to stay intravascular. Future studies should address the clinical obstacles found in this study, and less-invasive alternatives to determine MSFP should be further explored.

## Data Availability

The original contributions presented in the study are included in the article/Supplementary Material; further inquiries can be directed to the corresponding authors.
