# Supplementary material for: Feasibility to estimate mean systemic filling pressure with inspiratory holds at the bedside
Source: Front Physiol. 2022 Nov 29;13:1041730. doi: 10.3389/fphys.2022.1041730 (PMC9745184; doi:10.3389/fphys.2022.1041730)
Supplement: Supplementary file 1 [file DataSheet1.pdf]

Supplemental material 1.

CONSORT
TRANSPARENT REPORTING of TRIALS

CONSORT 2010 Flow Diagram

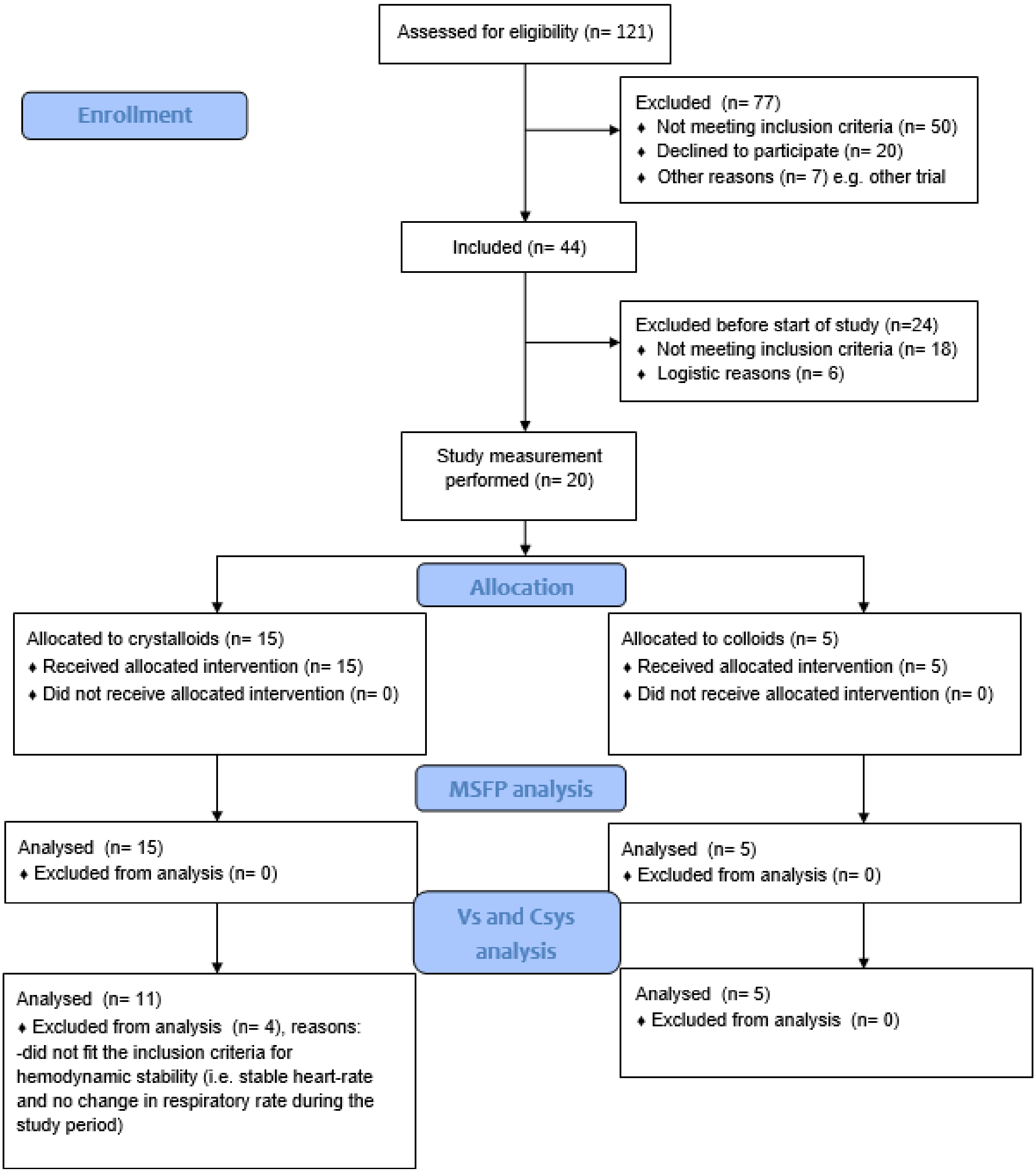

Supplemental Material 2. Characteristics study measurements

	Start study	End study
Temperature (°C)	36.1 +/- 0.44	36.2 +/- 0.52
Fluid balance (mL)	1890 (IQR 1769-2685)	2251 (IQR 1881-3020)
Hb (mmol/L)	7.53 +/- 1.01	7.23 +/- 0.97
pH	7.39 +/- 0.05	7.41 +/- 0.05
Lactate	1.31 +/- 0.61	1.29 +/- 0.52
FiO2 (%)	30 (IQR 30-40)	
Tidal volumes (mL)	528.14 +/- 57.1	
Tidal volume (ml/kg) TBW	6.0 +/- 0.96	
Tidal volume (ml/kg) IBW	6.7 +/- 0.7	
PEEP (cmH2O)	5 +/- 0	
Plateau pressure (cmH2O)	11.6 +/- 2.6	
Respiratory rate (per minute)	12 (IQR 12-14.5)	
Sufentanil	11 (60%)	
Mcg/hr	40 (IQR 20-41)<br>35 +/- 12	
In mcg/kg/hr	0.45 (0.25-0.48)<br>0.40 +/- 0.15	
In mcg/kg/min	-	
Propofol	20 (100%)	
Mg/hr	400 (IQR 303-400)<br>368 +/- 53.50	
in mg/kg/hr	4.48 (IQR 3.48-4.80)<br>4.21 +/- 0.80	
In mg/kg/min	0.08 (IQR 0.06-0.08)<br>0.07 +/- 0.01	
Noradrenalin	20 (100%)	
Mcg/hr	325 (IQR 300-500)<br>368.0 +/- 176.5	
Mcg/kg/hr	3.71 (IQR 1.18-5.31)<br>4.18 +/- 2.10	
Mcg/kg/min	0.06 (IQR 0.05-0.09)<br>0.07 +/- 0.035	

°C: degrees Celsius. mL: milliliters. Hb: haemoglobin. mmol=millimol, L=liter, FiO2: fraction of inspired oxygen. IBW: ideal body weight. TBW: total body weight. Medication dosage calculated with total body weight. Continuous data are presented as mean with standard deviation (+/-) or median with inter quartile ranges (IQR 25th-75th). Categorical data are presented as frequencies with percentages.

Supplemental Material 3. Delta MSFP, stressed volume and compliance.

Crystalloid (n=15)									
	Bolus1	Bolus2	Δ MSFP T=1 – T=0	Δ MSFP T=2– T=1	Csys1	Csys2	Vs1	Vs2	Δ / Δ MSFP
MSFP10	100	500	-2.28	8.89	-43.86	56.24	-879.82	1628.23	-3.90
MSFP12	100	400	2.74	1.57	36.50	254.78	530.66	4104.46	0.57
MSFP13	100	705	-1.01	5.21	-99.01	135.32	-1614.85	2912.02	-5.16
MSFP14	100	500	-1.06	2.34	-94.34	213.68	-1544.34	3997.86	-2.21
MSFP16	100	500	0.85	8.28	117.65	60.39	2337.65	1699.88	9.74
MSFP17	100	500	0.99	4.02	101.01	124.38	2241.41	3259.95	4.06
MSFP18	*100*	*500*	*3.48*	*6.90*	*28.74*	*72.46*	*710.63*	*2292.03*	*1.98*
MSFP19	*100*	*500*	*10.29*	*-2.07*	*9.72*	*-241.55*	*276.97*	*-6384.06*	*-0.20*
MSFP20	100	500	0.74	5.56	135.14	89.93	2185.14	1954.14	7.51
MSFP23	*100*	*500*	*0.67*	*1.02*	*149.25*	*490.20*	*3529.85*	*12093.14*	*1.52*
MSFP25	100	500	3.20	7.84	31.25	63.78	543.44	1586.73	2.45
MSFP26	*100*	*250*	*0.16*	*0.28*	*625.00*	*892.86*	*15175.00*	*22169.64*	*1.75*
MSFP28	100	500	1.29	8.99	77.52	55.62	2054.26	1899.89	6.97
MSFP37	100	500	1.79	6.14	55.87	81.70	1213.41	2103.76	3.42
MSFP44	100	400	3.36	2.83	29.80	141.13	691.18	3247.34	0.84
Colloids (n=5)									
MSFP36	100	300	3.01	8.96	33.26	33.49	655.11	922.14	2.98
MSFP38	100	500	9.39	7.84	10.65	63.78	361.24	2379.46	0.83
MSFP39	100	250	1.90	5.65	52.63	44.25	1221.58	1232.74	2.97
MSFP41	100	500	3.94	11.40	25.38	43.86	662.69	1597.37	2.89
MSFP42	100	500	2.48	5.74	40.32	87.11	1042.34	2621.08	2.31

Δ MSFP = delta MSFP. Δ / Δ MSFP = second delta/first delta. Italics: 4 patients that did not fit the delta MSFP, Csys and Vs hemodynamic stability criteria: MSFP 18, MSFP 19, MSFP23 and MSFP 26. Csys: compliance. Vs: stressed volume. MSFP: mean systemic filling pressure.

Supplemental material 4. Clinical feasibility

**MSFP derived with inspiratory holds method**	**Clinical implementation in ICU**	**Rationale**	**Potential solution**
Restricted to fully sedated and mechanically ventilated patients	+/-	Requires a level of sedation higher than standard of care in ICU	Inspiratory holds with smaller incremental pressures
		Not all patients in ICU are mechanically ventilated	
Restricted to patients with continuous and accurate CO and CVP measurements	+	Patients in which the treating ICU consultant is interested in MSFP usually have a central venous catheter to receive vasoactive drugs	
		The majority of patients in ICU have an arterial catheter for ABP monitoring, a CO measurement device can be connected	
		For MSFP,Vs and Csys calculation CO trends are sufficient. For RVR absolute values are required.	
Restricted to patients whom are hemodynamically stable for at least 4- 5 minutes	+	For most patients achievable	Inspiratory holds with smaller incremental pressures
Duration of Vs and Csys measurements	+/-	For a Vs or Csys measurement two MSFP measurements are required (=at least 10 minutes)	
Assumption that 100% of fluid administered remains in the intravascular compartment for the duration of the measurement	?	Crystalloids are the default fluids in the study ICU. We found a difference between colloids and crystalloids.	Future studies should aim to find the correct type of fluid (colloid/crystalloid) for Csys and Vs calculation
Hazardous?	?	Not known; only speculative	
		In this study in 50% of patients the fourth hold was withheld because of a too large decrease in MAP. However, three holds seem sufficient.	Inspiratory holds with smaller incremental pressures
		In the study hospital, executing inspiratory holds is restricted to ICU consultants only	
Number of people needed for one MSFP measurement	+	In the present study the inspiratory holds were manually executed	Computerized MSFP measurement

ABP: arterial blood pressure. CO: cardiac output. CVP: central venous pressure. Csys: compliance.
ICU: Intensive Care Unit. Vs: stressed volume. MSFP: mean systemic filling pressure.

Supplemental material 5.

	Bolus2	Δ MSFP<br>T=2– T=1	Δ MSFP<br>T=2– T=1<br>Corrected
Crystalloid (n=15)			
MSFP10	500	8.89	8.89
MSFP12	400	1.57	1.96
MSFP13	705	5.21	3.70
MSFP14	500	2.34	2.34
MSFP16	500	8.28	8.28
MSFP17	500	4.02	4.02
MSFP18	*500*	*6.90*	*6.90*
MSFP19	*500*	*-2.07*	*-2.07*
MSFP20	500	5.56	5.56
MSFP23	*500*	*1.02*	*1.02*
MSFP25	500	7.84	7.84
MSFP26	*250*	*0.28*	*0.56*
MSFP28	500	8.99	8.99
MSFP37	500	6.14	6.14
MSFP44	400	2.83	3.54
15 patients, mean +/-		4.52 +/- 3.43	4.51 +/- 3.36
11 patients, mean +/-*		5.61 +/-2.68	5.57 +/- 2.63
Colloids (n=5)			
MSFP36	300	8.96	14.93
MSFP38	500	7.84	7.84
MSFP39	250	5.65	11.30
MSFP41	500	11.40	11.40
MSFP42	500	5.74	5.74
Mean +/-		7.92 +/- 2.40	10.24 +/- 3.55

A post-hoc analysis to correct for the patients who did not receive the total of 500 mL of fluids during the second fluid bolus. The post-hoc analysis was performed by dividing the planned amount of fluid administered (=500 mL) by the actual amount of fluids administered (in mL), times the delta MSFP.

For example: MSFP 12, planned = 500 mL. Provided = 400 mL. 500/400= 1,25. Delta found = 1.57 mmHg. Corrected delta MSFP = 1.57 * 1.25 = 1.96.

Δ MSFP = delta MSFP. Italics: 4 patients that did not fit the delta MSFP, Csys and Vs hemodynamic stability criteria: *MSFP 18, MSFP 19, MSFP23 and MSFP 26*. Csys: compliance. Vs: stressed volume. MSFP: mean systemic filling pressure.

Utilizing all 20 patients, mean delta MSFP crystalloid 4.51 sd 3.36 and mean delta MSFP colloid 10.24 sd 3.55, using the independent t-test, $p < 0.01$

Utilizing the 16 patients who fulfilled the hemodynamic stability criteria, using the independent t-test, mean delta MSFP crystalloid 5.57 sd 2.63 and mean delta colloid 10.24 sd 3.55, $p = 0.01$

Supplemental material 6.

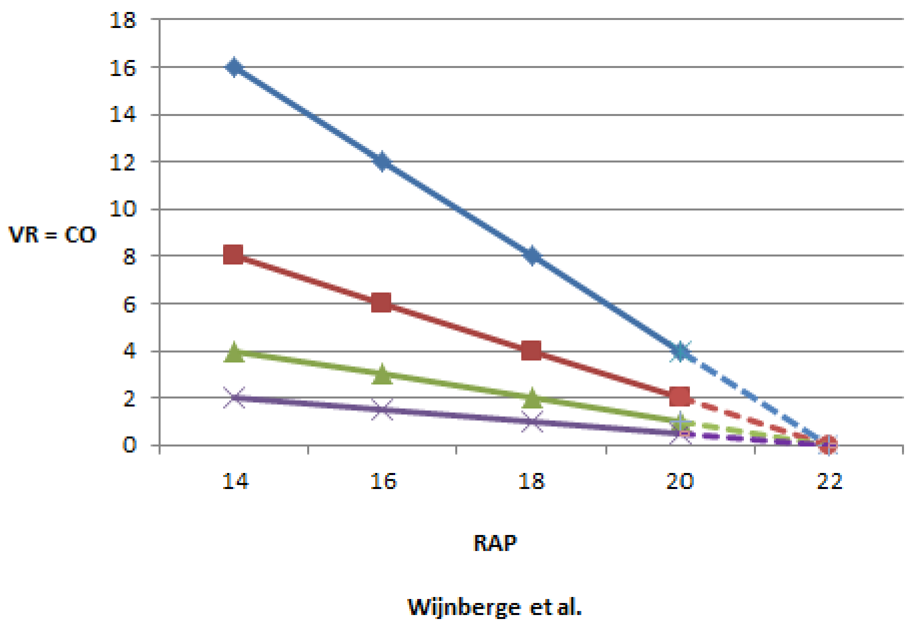

A correct absolute cardiac output (CO) value is needed for the slope of the line, defining the resistance to venous return (RVR). However if the uncalibrated CO values are consistent too high or too low (i.e. if the trend is correct), the intersection point will be similar, resulting in a similar mean systemic filling pressure (MSFP), compliance (Csys) and stressed volume (Vs). RAP: right atrial pressure.
